# Preference for Male Traits Differ in Two Female Morphs of the Tree Lizard, *Urosaurus ornatus*

**DOI:** 10.1371/journal.pone.0101515

**Published:** 2014-07-17

**Authors:** Matthew S. Lattanzio, Kevin J. Metro, Donald B. Miles

**Affiliations:** Department of Biological Sciences, Ohio University, Athens, Ohio, United States of America; Arizona State University, United States of America

## Abstract

Non-random female mating preferences may contribute to the maintenance of phenotypic variation in color polymorphic species. However, the effect of female preference depends on the types of male traits used as signals by receptive females. If preference signals derive from discrete male traits (i.e., morph-specific), female preferences may rapidly fix to a morph. However, female preference signals may also include condition-dependent male traits. In this scenario, female preference may differ depending on the social context (i.e., male morph availability). Male tree lizards (*Urosaurus ornatus*) exhibit a dewlap color polymorphism that covaries with mating behavior. Blue morph males are aggressive and defend territories, yellow males are less aggressive and defend smaller territories, and orange males are typically nomadic. Female *U. ornatus* are also polymorphic in dewlap color, but the covariation between dewlap color and female behavior is unknown. We performed an experiment to determine how female mate choice depends on the visual and chemical signals produced by males. We also tested whether female morphs differ in their preferences for these signals. Female preferences involved both male dewlap color and size of the ventral color patch. However, the female morphs responded to these signals differently and depended on the choice between the types of male morphs. Our experiment revealed that females may be capable of distinguishing among the male morphs using chemical signals alone. Yellow females exhibit preferences based on both chemical and visual signals, which may be a strategy to avoid ultra-dominant males. In contrast, orange females may prefer dominant males. We conclude that female *U. ornatus* morphs differ in mating behavior. Our findings also provide evidence for a chemical polymorphism among male lizards in femoral pore secretions.

## Introduction

Female preference may contribute to the maintenance of morphological variation in species where males exhibit discrete phenotypes [1], [2]. Because females rely on multiple signals to assess potential mates [3], the selective consequences of female behavior depends in part on the types of male traits used as signals to inform preference. In polymorphic species, female preference signals may be based on discrete traits that are correlated with morph genetic differences or condition-dependent ornaments. Discrete sexual signals have the potential to lead to rapid fixation of female preference to a single or multiple male morphs [4], [5]. In contrast, use of condition-dependent male traits as signals for female preference may not favor assortative mating. The importance of condition-dependent or discrete male traits for female preference may differ with respect to the social context a female experiences (i.e., availability of each male morph). Understanding the contribution of different male traits to female preference across different social contexts may provide insight into the role of female preference in the maintenance of morphological variation.

Species exhibiting a discrete color polymorphism within a population present an excellent opportunity to evaluate the use of discrete and condition-dependent male traits in female preference. Color polymorphism occurs in a diverse range of taxa, and color differences are assumed to reflect alternative ecological [6] or reproductive [2], [7] strategies. In the latter case, color morphs are associated with phenotypic syndromes that integrate physiological, morphological, and behavioral traits that influence reproductive success [8], [9]. Multiple factors have been implicated in the maintenance of color morphs and include negative frequency-dependent selection [7] or spatial variation in selection. However, the role of mating preferences in the maintenance of discrete phenotypes is gaining support [1], [10]–[13].

In several lizard species, discrete male dewlap color differences are associated with alternative reproductive tactics [7], [14]–[16], and color morphs differ in behaviors that affect their reproductive success [2]. Male ornate tree lizards (*Urosaurus ornatus*) exhibit a color polymorphism that involves a solid blue, yellow, or orange dewlap linked with differences in aggression and resource-holding potential [8]. Blue males aggressively defend territories, yellow males exhibit satellite behavior or defend territories, and orange males are nomadic. Male tree lizards also possess two additional traits that may influence female preference. First, all males express a blue ventral patch on their abdomen. The size and color components of this patch vary continuously among individual males [17], [18], suggesting that expression of this trait may be condition-dependent [19]. Moreover, ventral patch size has a positive correlation to traits that signal resource-holding potential, such as bite force [17]. Second, individual males may differ in body condition (residual mass per unit body size). Body condition is an estimate of individual energy reserves [20], and may convey information regarding a males’ ability to succeed in agonistic contests, defend resources, or survival potential [20]. Because yellow, orange and blue males overlap in snout-vent length [17], body condition may convey information about male quality uncorrelated with the color signal. Differences in male dewlap color, ventral patch size, and body condition may all convey information to a female tree lizard regarding mate quality.

Female preferences may differ by social context given the costs and benefits of mate choice [5], [21]. For example, although male dewlap color is associated with variation in aggressive behavior and resource holding potential [22], female tree lizards may risk injury by associating with an aggressive male [23]. However, non-aggressive or subordinate males may be unable to defend a territory and access to nest sites, which may favor a sneaker strategy. Female tree lizards may evaluate both discrete (dewlap color) and condition-dependent (ventral patch size and color, and body condition) signals associated with social dominance to choose mates in these contexts. In species like *U. ornatus*, female preference may also be influenced by female morph [21], [24], [25]. If fitness costs associated with mate choice differ between female morphs, then their mating preferences may also differ. Jointly, context-dependent and morph-specific female preferences may induce variability to preference functions and their contribution to male differences in polymorphic systems [21], [24], [26].

The results of multiple studies also demonstrate the importance of male chemical signals for intraspecific communication in lizards [27]–[29]. In many lizard species, males exude femoral pore secretions. The compounds present within these secretions may provide information on aspects of male quality, such as immunocompetence [27] and health [30]. In addition, females may also be capable of discriminating among male color morphs by femoral pore secretions alone [31]. Previous work hints that male *U. ornatus* femoral pore secretions play a role in intraspecific communication [32], but the contribution of these secretions to female preference is unclear.

Here we evaluate the contribution of discrete and condition-dependent male traits to female preference in *U. ornatus* in order to gain insight into the role of female preference in maintaining phenotypic variation in polymorphic species. If female preferences are fixed to a specific morph, then females should exhibit assortative behavior towards that morph when it is present. The influence of male condition-dependent traits on mate choice is predicted to be low or absent in this scenario. Alternatively, if female preferences are not fixed, females may supplement information from fixed signals and use variation in condition-dependent male traits as preference signals. Use of condition-dependent male traits may be enhanced in social contexts where the available males differ in dewlap color from the female.

Next, we conducted an experiment to test the hypothesis that female *U. ornatus* are capable of discriminating among male morphs using chemical signals alone. We then compared patterns of female behavior in response to male visual (dewlap color) and chemical (femoral pore secretion) signals. Similar non-random patterns in female behavior towards male morphs in both modalities would provide evidence that chemical and dewlap color cues provide complementary information to receptive females.

## Materials and Methods

### Ethics Statement

Experiments were approved by the Ohio University Institutional Animal Care and Use Committee (protocol #R06–07) and the Arizona Department of Fish and Game (permit #SP792912).

### Capture Methods and Lizard Morphology

Adult male and non-gravid female *U. ornatus* were collected at the Appleton-Whittell Research Ranch in Santa Cruz County, Arizona (31° 35.428 N, 110° 30.388 W) during July 2011, which falls within the breeding season of *U. ornatus* at our study site. Lizards were sexed based on the presence of enlarged post-anal scales (males). The frequency of each *U. ornatus* color morph varies by population. At our study site, male *U. ornatus* lizards have a solid blue (51%), yellow (20.4%), or orange (18.4%) dewlap (Figure S1). Female *U. ornatus* have either a solid yellow (48.1%) or orange (40.7%) dewlap (Figure S1). Females without dewlap coloration (11.2%) and bicolor (10.2%) male morphs also occur, but at low encounter frequencies. We excluded these morphs in our study. The morph (dewlap color) of each lizard was recorded upon capture. Body size (snout-vent length, SVL) was measured to the nearest 0.5 mm, and mass was recorded to the nearest 0.1 gram using a Pesola scale (Pesola AG, Baar, Switzerland).

Male *U. ornatus* possess a blue ventral patch that is exposed to females during courtship [33]. The size of this patch varies among males [17]. Digital images of the ventral patch were obtained by scanning the ventral surface of each individual male lizard in a Canon LiDE flatbed scanner (Canon, Melville, NY, USA). Prior to scanning, we warmed each male lizard under a light (75 W) until it reached a body temperature of ca. 34–36°C to ensure full expression of its blue ventral patch [34]. The size (in pixels) of this patch was then estimated using the polygon tool in the program ImageJ version 1.44 p [35]. We calculated ventral patch size (mm^2^) from these polygons. A ruler placed on the platen served as a reference for converting patch size measurements.

### Color Measurements

Reflectance data from the dewlap and ventral patches of each male lizard were measured following established methodology [36]–[39]. We used an Ocean Optics USB2000-UV-VIS spectrometer with a pulsed fiber-optic light source (Ocean Optics Worldwide, Dunedin, FL, USA) to record reflectance data. All reflectance measurements were made relative to a WS-1 white standard (Ocean Optics, Dunedin, FL, USA) and to darkness. We recorded reflectance data spanning the visual range of lizards (320–700 nm) [40], in 0.3 nm increments. We recorded three reflectance measurements consisting of an average of 20 readings at rostral, central, and caudal portions of each color patch (integration time = 295 ms, boxcar correction = 10) (refer to Figure S1 for photographs of each morph illustrating dewlap and ventral color patch variation among male lizards). For each measurement, we held the probe at a 45° angle above the color patch. A non-reflective opaque tube was affixed to the probe to block ambient light.

Reflectance data were averaged over these three regions for each patch to ensure that we captured the color variation present across the entire surface of a patch. These reflectance data were further divided into visible (vis-, 400–700 nm) and ultraviolet (uv-, 320–399 nm) ranges [41]. Preliminary analyses suggested that only blue morph males reflect >5% in the uv-range in their dewlaps (Figure S2). Male ventral patches also varied little in their uv-range percent reflectance (Figure S2) and the color components extracted from these data (i.e., 19 of 29 males expressed same chromatic values, see below for color extraction procedure). We retained vis-range reflectance data from the ventral color patches for further analyses.

We extracted vis-range values each of brightness, hue, and chroma following methods in [36] implemented using the program Spectre (https://pantherfile.uwm.edu/pdunn/www/Spectre/Spectre.html). We applied a principal component analysis (PCA) to condense the resulting hue, chroma, and brightness values into a single color score based on extraction of the scores from the first principal component axis (PC1) [39], [42]. This axis accounted for between 76.1 and 76.4% of the variation in visible-range color properties of the dewlap and ventral patches, respectively (Table 1).

**Table 1:** Component loadings of the first axis of two Principle Components Analyses (PCAs) performed on dewlap and ventral color patch color properties, respectively.

Male color patch	Variance explained (%)	Eigenvalue	Factor loadings		
			Brightness	Hue	Chroma
Dewlap color patch	76.1	1.82	0.679	−0.66	−0.322
Ventral color patch	76.4	2.29	0.572	0.519	−0.635

We used PCA to describe dewlap and ventral patch hue, chroma, and brightness as an independent color score (based on the first PCA axis, PC1) [36]. Factor loadings indicate the relative direction and magnitude of contribution by each color component to a score.

### Female Preference for Male Visual Signals

Our first experiment assessed the preferences of female *U. ornatus* for the different male morphs. Individual male (n = 29) and female (n = 19) lizards were held in separate 5.7 liter covered terraria (27.9×17.8×12.7 cm, l×w×h, Frey Scientific, Nashua, NH, USA) at a field laboratory throughout the duration of this experiment. We maintained lizards on a 15∶9 h (light:dark) photoperiod. Temperatures fluctuated with ambient conditions. Lizards were offered two calcium gut-loaded crickets (*Acheta domestica*) daily and provided water *ad libitum*. Each enclosure had paper affixed to its sides to prevent lizards from interacting prior to experimentation.

Males were grouped by morph (yellow [n = 8], blue [n = 9], or orange [n = 12]; hereafter Y, B, or O). Female yellow (n = 9) and orange (n = 10) morphs were randomly exposed to one of three polymorphic male dyads, Y-O, B-O, or Y-B, for their first trial [43]. Following that trial, each female was then randomly assigned to one of the remaining two dyads. Females were allowed 24 hours to rest between successive trials. Pairs of males were chosen at random from each morph category. We also attempted to minimize body size differences in each dyad (mean ± SD male size difference: SVL, 1.4±1.2 mm; mass, 0.3±0.2 g).

We used an experimental arena (total size: 1×0.3×0.3 m, l×w×h) lined with a 1-cm layer of sand for observing the behavior of females during exposure to males. We placed males into two chambers (0.3×0.3 m, l×w) on opposite sides of this arena. Males were prevented from interacting with a female by clear plastic sheets secured to the arena floor and walls, and each chamber also had covers to prevent airflow between chambers. Because male *U. ornatus* femoral pore secretions are non-volatile [28], these dividers and lids ensured that female assessment of the males only involved visual signals. Moreover, while the two males could see each other in the arena, results from staged contests between male *U. ornatus* indicate that they only initiate display behavior with each other when in close proximity (ca. <20 cm, Lattanzio & Miles, unpublished data). We designed our experimental arena so that males are always ≥40 cm apart from each other, and we did not observe any male-male interactions during this experiment. Equal heat and lighting was maintained throughout this arena with the aid of suspended 75 W lamps. We used sand as a substrate in the arena and replaced this sand at the conclusion of each trial. At the beginning of a trial we placed a female lizard into the center of the arena and oriented perpendicular to the two male chambers. Each female was allowed 5 minutes to acclimate to the arena, at which point we simultaneously introduced one male into each side chamber. We randomized the side a male morph was placed for each trial. We allowed females to observe the males for a period of eight hours, beginning between 0800 and 1000 h. This study design ensured that we observed lizards throughout their daily activity period (ca. 0800–1800 h), thereby providing a more accurate assessment of female preference [44]. One observer (KJM) recorded the position of the focal female at 30 min intervals from behind a blind. Each trial resulted in 16 observations. Female visits were scored based on their proximity to each male. We considered a male to be visited if the female was within 10 cm of his chamber, and assigned a score of zero if a female was >10 cm from either male. At the end of each trial we tallied the number of visits toward each male. Tree lizards are sedentary, ambush predators and move infrequently throughout the day (Lattanzio, personal observation). Female *U. ornatus* do not shuttle even during thermoregulatory behavior, and will spend more time near a male if they exhibit an actual preference for that male in the wild (Miles, unpublished data). Thus, the location of a female at one of our 16 observation points reflects both her position for most (if not all) of the previous 30 min and, depending on her position in the arena, her preference for a male.

### Female Preference for Male Chemical Signals

We conducted a second experiment to determine whether female *U. ornatus* are capable of discriminating among male color morphs using their femoral pore secretions. For these trials, we used the same females from the visual choice experiment. We obtained chemical secretions from a second sample of male lizards captured from the same population. Females did not interact with these males prior to or during experimentation. Upon completion of the visual experiment, we returned all females to their individual enclosures for 10 days. Food (crickets) and water were provided daily. Chemical preference trials were then conducted within a different arena (total size: 1.22×0.15×0.25 m, l×w×h). We suspended 75 W lights above this arena to maintain similar heat and lighting conditions throughout this arena. We used sand as a substrate in this experiment and replaced it between all trials.

We used small plastic trays (80×50×14 mm, l×w×h; Sigma-Aldrich, St. Louis, MO, USA) as tiles in this experiment. The same male dyads were used in this experiment to facilitate comparisons with the visual experiment. We exposed each female to two of the three dyads in a random order. Dyad exposure was randomized so females did not necessarily experience the same two male dyads in this experiment that she experienced in the visual experiment. Femoral pore secretions were collected from each male morph using sterile cotton tipped applicators (Medline Industries, Mundelein, Illinois, USA). To collect pore secretions, we first dipped an applicator into distilled water and then rubbed on the proximal portion of a males’ hind limbs (location of femoral pores) for 60 seconds. Applicators were labeled by morph and stored in individual plastic freezer bags within a larger bag (one per morph) at −20°C in a freezer [45]. To apply a scent, we randomly selected an applicator from the appropriate bag and swabbed the tile surface for a period of 30 seconds, immediately prior to experimentation. Nitrile gloves were worn during collection and application procedures. We placed two scented tiles in the arena so that each tile was approximately 5 cm from its end of the arena. At the start of a trial, we placed a female into the center of an arena, perpendicular to the direction of either tile. The position of the female in the arena was recorded after 20 minutes, and every 10 minutes thereafter, for a total of 90 minutes (eight readings total). Female visits were scored based on their proximity to a tile. A score of zero was assigned if a female was observed >5 cm from either tile. We discarded used tiles and applicators after each trial. One observer (MSL) recorded female positions during this experiment. At the end of each trial we tallied the number of visits toward each male.

### Statistical Analysis

Analysis of variance (ANOVA) was used to test whether male morphs differ in dewlap color. We found no relationship between male ventral patch size and ventral patch color score (*r* = 0.065, *t* = 0.34, df = 27, *P* = 0.737). We therefore applied separate analyses of covariance (ANCOVAs) to test whether the male morphs differ in ventral patch size or color, respectively. We used body size (SVL) as a covariate in both models. A homogeneity of slopes test indicated no interaction between male morph and SVL in either model (both *P*>0.1). We used the coefficient of variation (i.e., standard deviation/mean) to describe relative variation in ventral patch size among males of each morph [46].

Fisher’s exact tests were used to evaluate whether yellow or orange females exhibited a bias towards either side of the arena, independent of male morph, across the first and second trials for each female. We used a poisson regression to determine whether female morphs differed in mating preferences (number of visits to each male). In this model we included four factors accounting for the interaction between female morph, male dyad, and each male trait (morph, body condition, ventral patch size, or ventral patch color). Body condition was calculated as the residuals of a regression predicting mass from SVL [20]. A significant result associated with any of these predictors would suggest that female morphs differ in their preferences for male lizards based on the trait considered. Ventral patch size and color were treated as separate predictors because these patch properties are uncorrelated (*F*_1,27_ = 0.3, *r* = 0.03, *P* = 0.872).

We also used Fisher’s exact tests to investigate side bias and a poisson regression to evaluate the number of visits by females to each morph in each dyad in the chemical experiment. Finally, we used an ANOVA to compare the strength of preference (SOP) by each morph to the different male morphs between the visual and chemical experiments with an experiment×female morph×male dyad interaction as a factor. Here, SOP refers to the absolute value of the difference in number of visits to each male in the dyad by a female during a trial. A non-significant effect of the experiment×female morph×male dyad interaction provides evidence that male *U. ornatus* visual and chemical signals convey similar information to a receptive female. We included female identity as a covariate in this analysis.

Data were log_10_-transformed when necessary, and we used post-hoc pairwise comparisons to compare factors with >2 levels. We conducted all statistical analyses in r 3.02 [47] and SPSS 19 (SPSS Inc., Chicago, IL, USA).

## Results

### Morphological Variation among Male *U. ornatus* Lizards

**Figure 1:**
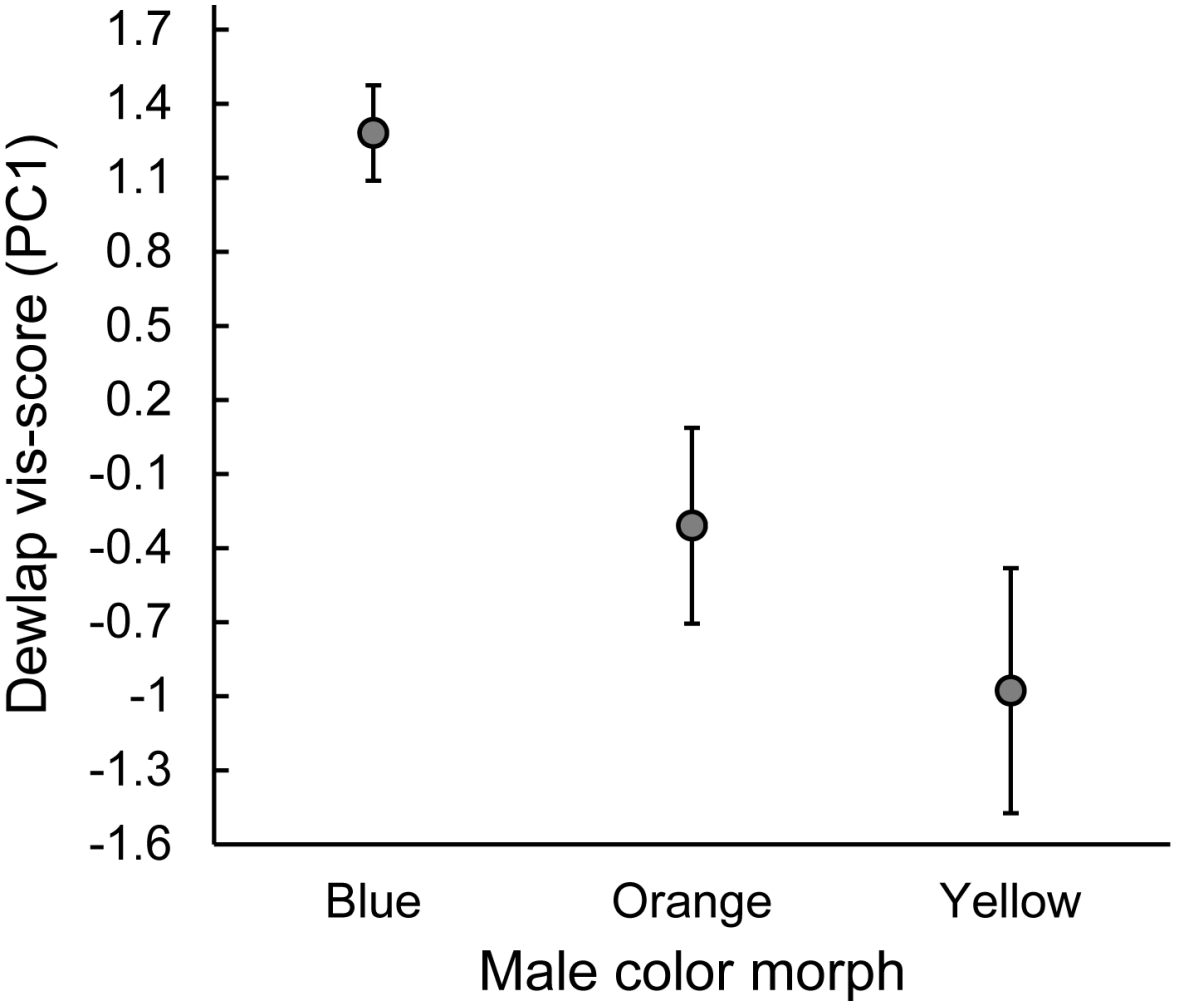
Dewlap color differences among male tree lizard (*Urosaurus ornatus*) morphs. Dewlap color refers to scores from the first principal component axis derived from hue, chroma, and brightness data collected from male dewlaps (n = 29 males, see Materials and Methods for color procedure). Male lizards are grouped by morph: blue, orange, or yellow (see Figure S1). Values are mean ±1.0 standard error (SE).

Male *U. ornatus* dewlap color scores differed among the three morphs (*F*_2,26_ = 8.23, *P* = 0.002). Blue males had brighter dewlaps than orange (pairwise comparisons, *P* = 0.015) and yellow males (pairwise comparisons, *P* = 0.002), but yellow and orange males did not differ (pairwise comparisons, *P* = 0.451, Figure 1). In contrast, morphs do not differ in ventral patch size (*F*_2,25_ = 1.59, P = 0.225; B males, 202.5±10.1 mm^2^; O males, 185.1±6 mm^2^; Y males, 185.2±6.3 mm^2^). Intra-morph variation in patch size was also similar (coefficient of variation, %: CV_B_ = 14.9%, CV_Y_ = 9.6%, CV_O_ = 11.2%). Ventral patch size does not covary with body size (*F*_1,25_ = 2.06, *P* = 0.163). Male morphs also exhibit similar ventral patch color scores (*F*_2,25_ = 0.47, *P* = 0.628). There is no relationship between ventral patch color score and body size (*F*_1,25_ = 1.21, *P* = 0.283).

### Female Preference for Male Visual Signals

Yellow and orange morph female *U. ornatus* did not bias their visits towards a particular side of the arena (yellow females, Fisher’s exact test, *P* = 0.729; orange females, Fisher’s exact test, *P* = 0.878). Yellow and orange morph female *U. ornatus* differed in their responses to male lizards with respect to male morph and ventral patch size only (female morph×male dyad×male morph, *χ* ^2^ = 26.32, df = 11, *P* = 0.006; female morph×male dyad×male ventral patch size, *χ* ^2^ = 21.97, df = 6, *P* = 0.001). In contrast, the two female morphs did not differ in their responses to male lizards with respect to male body condition and ventral patch color score (female morph×male dyad×body condition, *χ* ^2^ = 5.49, df = 6, *P* = 0.483; female morph×male dyad×ventral patch color score, *χ* ^2^ = 6.91, df = 6, *P* = 0.329).

**Figure 2:**
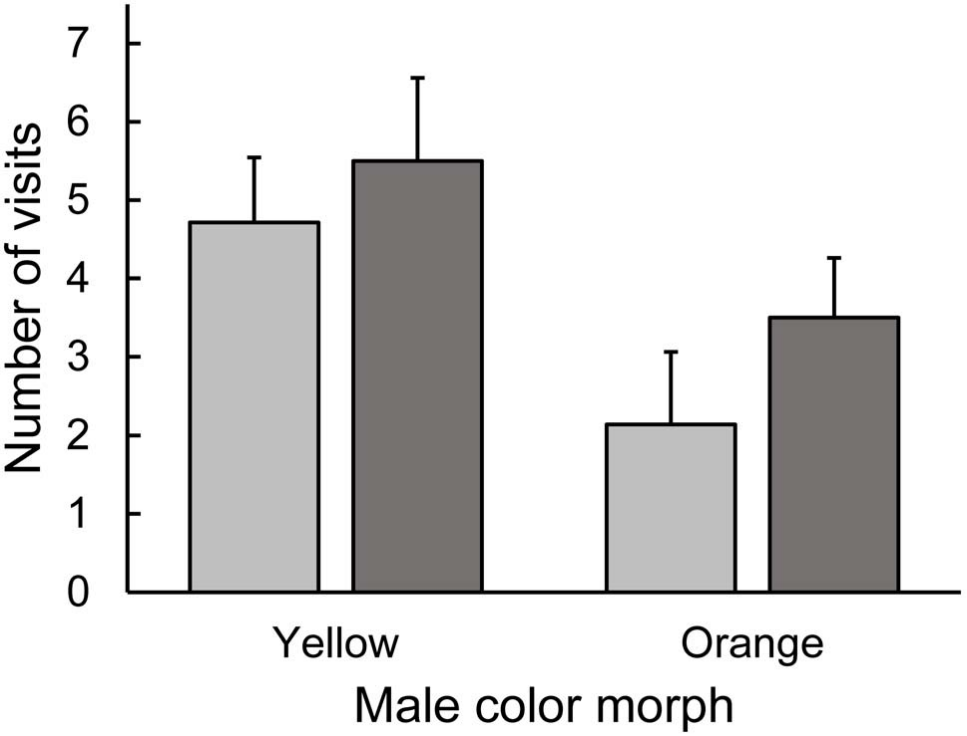
Preference of female *Urosaurus ornatus* when presented with orange and yellow males (visual experiment). Preference by yellow (light grey) and orange (dark grey) morph females refers to a greater number of visits towards one male over another and bars are mean+1.0 standard error (SE). Both yellow and orange females preferred yellow over orange morph males (see Results).

**Figure 3:**
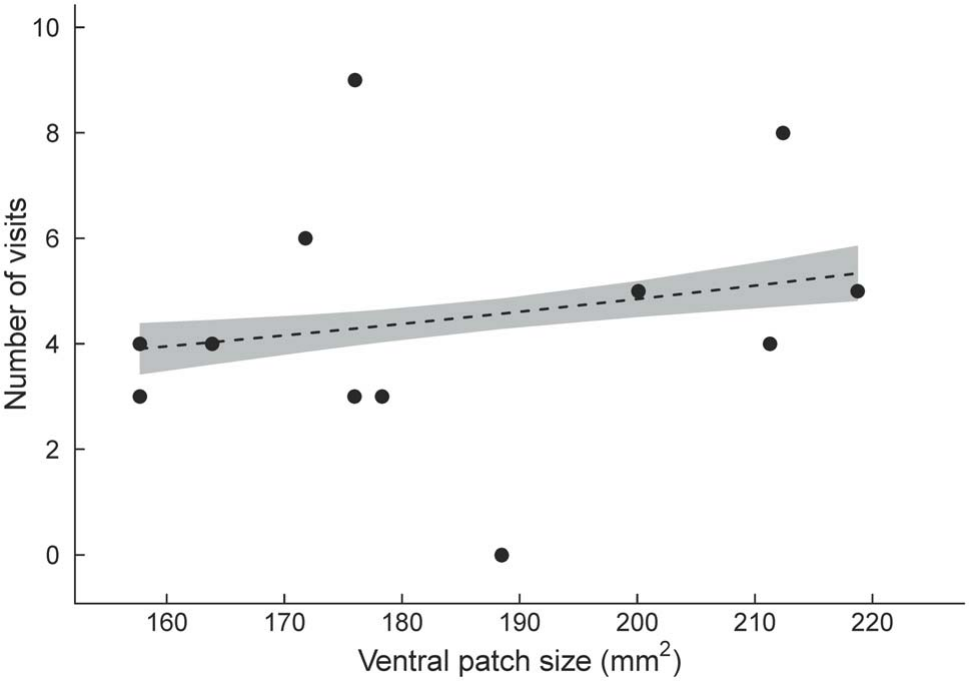
Relationship between male *Urosaurus ornatus* ventral patch size and orange female preference (visual experiment). Preference refers to the number of visits by orange females to males that differ in ventral patch size (mm^2^) in the Y-O dyad. The grey shaded region denotes 95% confidence intervals around predicted values from a poisson regression.

Both male morph and ventral patch size predicted the number of visits to a male by the two female *U. ornatus* morphs. Yellow and orange females behaved similarly in the Y-O dyad (pairwise comparisons, all *P*>0.07), with the exception that orange females visited yellow males more often than yellow females visited orange males (pairwise comparisons, *P* = 0.002). Both yellow and orange female morphs preferred yellow males in this dyad (pairwise comparisons, *P* = 0.041 and *P* = 0.016, respectively) (Figure 2). Moreover, orange females visited males with a larger ventral patch more often than males with a smaller patch in this dyad (*χ* ^2^ = 4.61, df = 1, *P* = 0.032) (Figure 3).

**Figure 4:**
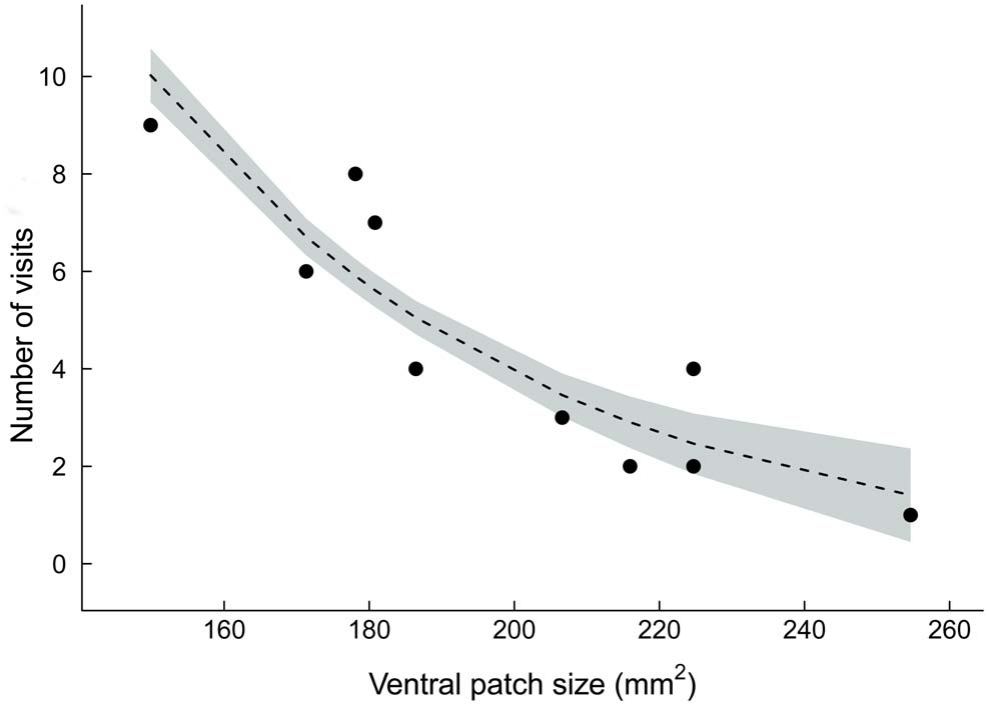
Relationship between male *Urosaurus ornatus* ventral patch size and yellow female preference (visual experiment). Preference refers to the number of visits by yellow females to males that differ in ventral patch size (mm^2^) in the B-O dyad. The grey shaded region denotes 95% confidence intervals around predicted values from a poisson regression.

Neither female morph exhibited a preference for a single male morph in the B-O dyad (pairwise comparisons, yellow females, *P* = 0.606; orange females, *P* = 0.077). However, yellow females visited males with smaller ventral patches more often than males with larger ventral patches in the B-O dyad (*χ* ^2^ = 7.66, df = 1, *P* = 0.006) (Figure 4). Moreover, both yellow and orange females visited orange males more often in the B-O dyad than in the Y-O dyad (pairwise comparisons, yellow females, *P*<0.001; orange females, *P* = 0.003). Both female morphs behaved similarly in the Y-B dyad and did not exhibit a preference for either male morph (pairwise comparisons, all *P*>0.07).

### Female Preference for Male Chemical Signals

A preliminary study revealed that female *U. ornatus* are able to discriminate between tiles scented with male femoral pore secretions and unscented (distilled water), based on differences in number of tongue-flicks towards each stimulus (χ^2^ = 5.33, df = 1, *P* = 0.021, n = 12 females [six orange, six yellow]). We found no evidence of side bias for either female morph in this experiment (yellow females, Fisher’s exact test, *P* = 0.864; orange females, Fisher’s exact test, *P* = 0.266).

**Figure 5:**
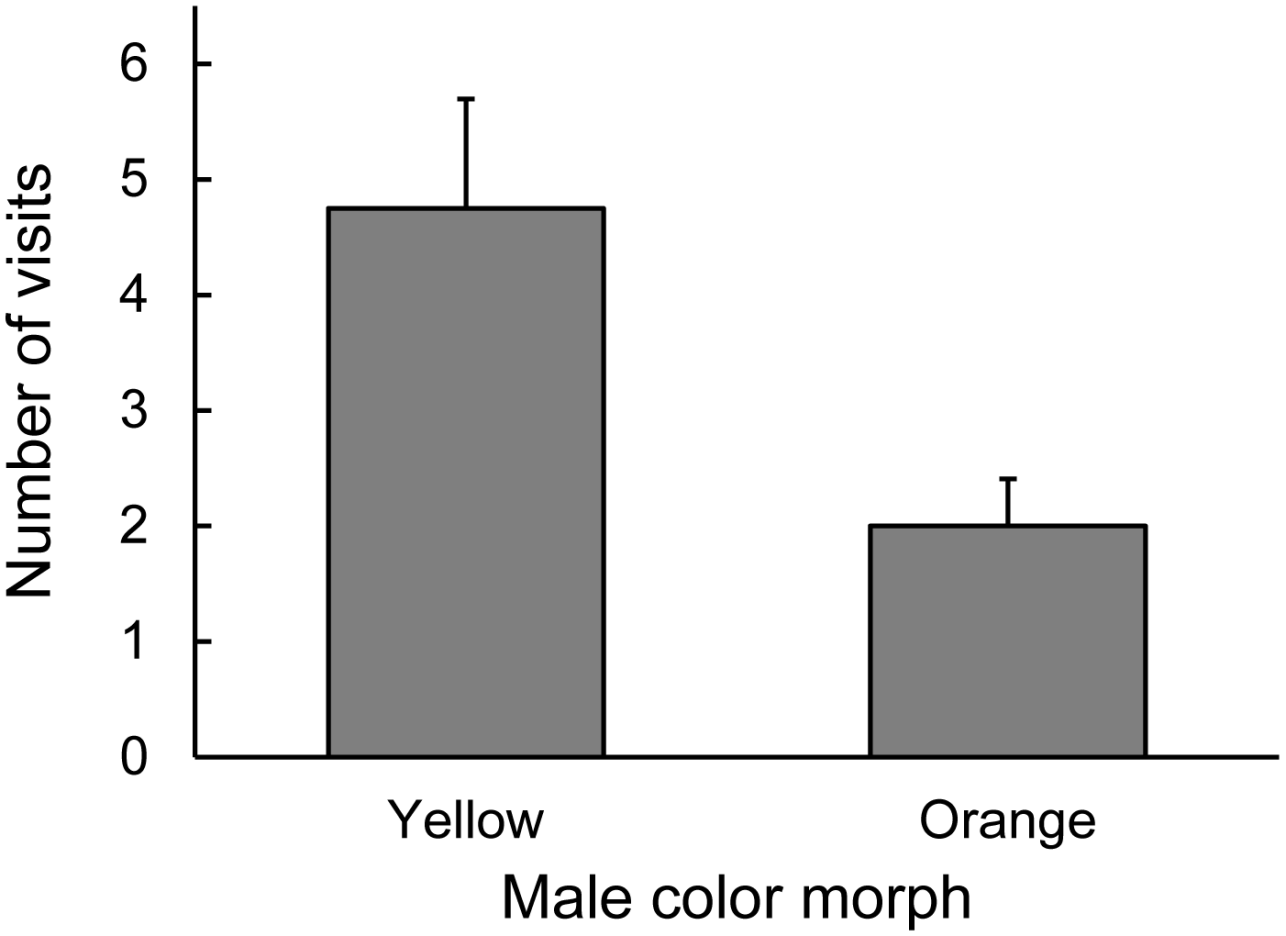
Yellow female *Urosaurus ornatus* preference for chemical signals of yellow and orange males. Preference refers to the number of visits by yellow females to either tile in the Y-O dyad. Bars are mean+1.0 standard error (SE). Yellow females preferred tiles scented with yellow male secretions (see Results).

Both female morphs exhibited similar mating preferences with respect to male morph in the chemical experiment (female morph×male dyad×male morph, *χ* ^2^ = 15.08, df = 11, *P* = 0.178). Yellow females preferred yellow males over orange males in the Y-O dyad (pairwise comparisons, *P* = 0.002) (Figure 5). In addition, yellow females exhibited a stronger preference for yellow males than orange females did for either male morph in this dyad (pairwise comparisons, *P* = 0.021 and *P* = 0.013 for yellow and orange males, respectively).

### Patterns of Female Behavior across Signal Modalities

**Figure 6:**
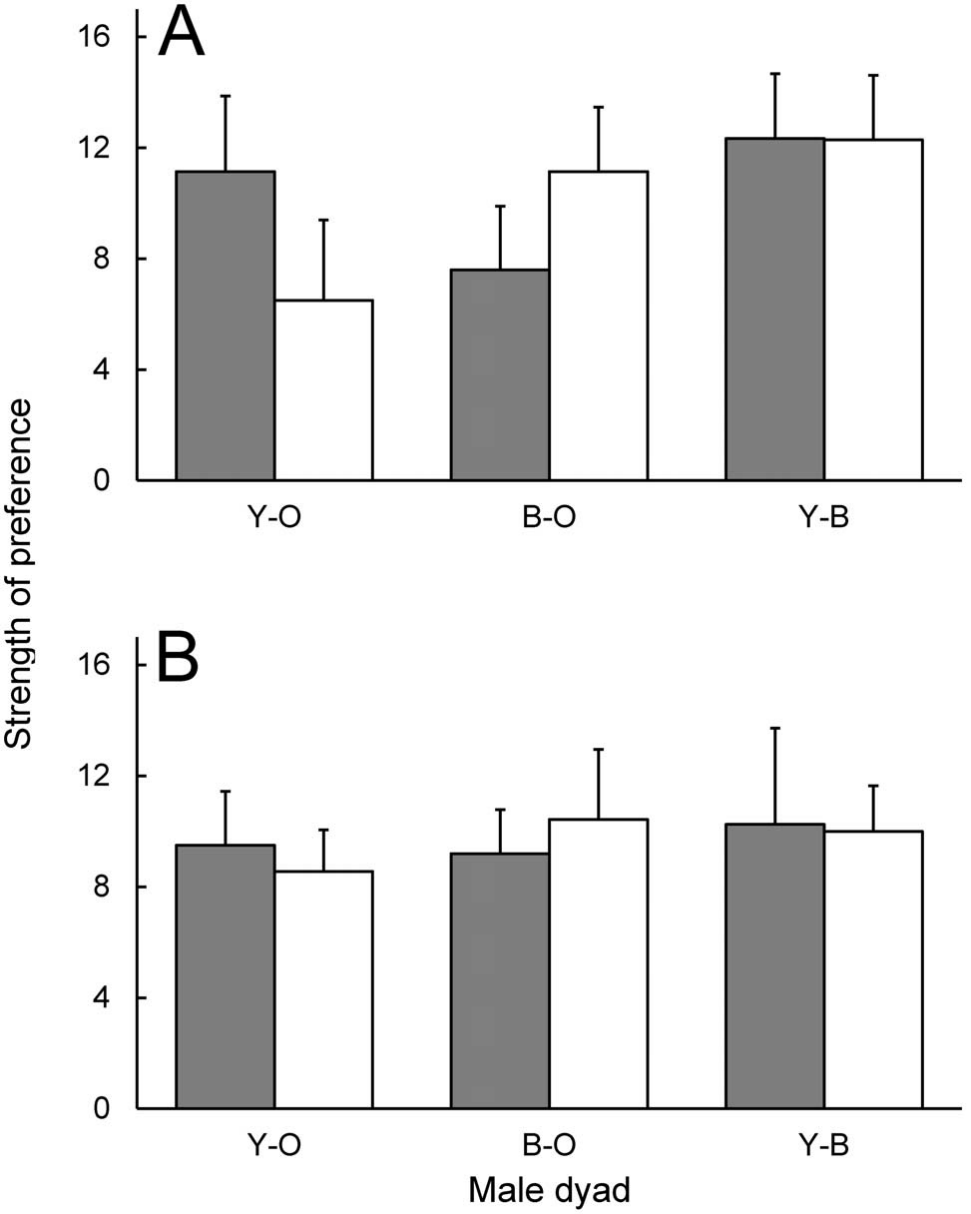
Strength of preference for male *Urosaurus ornatus* by the two female morphs. Strength of preference (absolute difference in number of visits) for both yellow (A) and orange (B) morph female *U. ornatus* in each dyad. Bars are shaded by experiment (grey: visual, white: chemical) and are mean+1.0 standard error (SE).

Yellow and orange females displayed a similar SOP for males in both experiments (experiment×female morph×male dyad interaction, *F*_11,63_ = 0.64, *P* = 0.791). In other words, male femoral pore secretions and dewlap color elicited similar behavioral responses by females with respect to their mating preferences (Figure 6). Repeated use of individual females did not affect female SOP (*F*_1,63_ = 2.3, *P* = 0.135). These results are also robust to weighting SOP by the total number of visits to both males in each trial (both *P*>0.43).

## Discussion

Our findings highlight heterogeneity in mate preferences by two female *U. ornatus* morphs for the three male morphs. Both yellow and orange female morphs had a preference for a specific male morph, but this preference was contingent on the pairing presented to the female. Both yellow and orange females preferred yellow males, but only over orange males. Orange females also exhibited a preference for males with a larger ventral patch (a continuous trait) in this dyad. In contrast, yellow females only used male ventral patch size signals when both males differed in dewlap color from the female (i.e., the B-O dyad). Although yellow and orange morph females behaved similarly in the chemical experiment, yellow females exhibited a stronger preference for yellow males over orange males. Overall, both female morphs behaved similarly towards male morphs across the visual and chemical experiments (see Figure 6), supporting that male morphs may have different femoral pore secretions. We conclude that female preferences in *U. ornatus* vary depending on male morph availability. Our work also provides some evidence for a chemical polymorphism among male *U. ornatus*.

In male *U. ornatus*, lizards with a larger ventral patch have a greater bite force, a trait linked with social dominance [17]. In support of our prediction, ventral patch size appeared to influence yellow females when offered a choice between a blue and an orange male. In *U. ornatus* and many other species, males defend territories and access to critical resources (e.g., oviposition sites). In *U. ornatus*, males with blue dewlaps are the dominant, highly-aggressive morph in resource contests [8]. However, females risk injury or mortality by associating with an aggressive phenotype [23], [48], [49], suggesting that aggressive males should not always be preferred mates [50]. For example, in three-spine sticklebacks (*Gasterosteus aculeatus*), highly-aggressive males often fail at mating attempts but less-aggressive males are able to mate repeatedly with the same female [51]. Likewise, female Japanese quail (*Coturnix japonica*) prefer males that lose over males that win competitive interactions [52].

Preference by yellow female *U. ornatus* for yellow over orange males in one social context and towards males with smaller ventral patches in a different context (i.e., choice between a blue and an orange male) suggests that yellow female *U. ornatus* may also avoid associating with highly-aggressive males [17]. Females preferring less-aggressive males may lose both direct (e.g., access to resources) and indirect (e.g., offspring sired by higher-genetic quality males) reproductive benefits [50]. However, because all three male *U. ornatus* morphs are capable of defending resources to a limited degree [53], preference for a less-aggressive male may provide a female with reproductive benefits (e.g., resource access) and minimal risk of injury during courtship.

When offered a choice between a yellow and an orange male, orange females preferred both yellow males and males with a larger ventral patch. It may be that orange females prefer the male that may be more likely to succeed in an agonistic encounter (i.e., males with a larger ventral patch) [17]. In other dyads, orange females did not exhibit a preference for males based on any traits considered. However, when offered a choice between a yellow and a blue male, the more-aggressive male morphs in *U. ornatus* (Lattanzio, unpublished data) [8], orange females tended to prefer the male having the larger ventral patch (75% of trials). This preference may provide reproductive benefits to an orange female (i.e., access to higher-quality nest sites) that may outweigh potential costs [50]. However, the extent to which males injure females during courtship (or other social interactions) in this species is unclear. Observations of male-female interactions in the wild or a laboratory setting are needed to add further insight into the costs and benefits of female choice in *U. ornatus*.

Female *U. ornatus* dewlap color is fixed at sexual maturity (Lattanzio unpublished data) [54], suggesting that other traits such as reproductive behavior may correlate with color differences among the morphs [55]. Our findings provide evidence that yellow and orange female morphs differ in signals used for mate choice. Female preference is likely dynamic in polymorphic species [21], and variable depending on the social context and morph of the female [24], [25]. Although yellow and orange morph female *U. ornatus* may use similar signals (dewlap color, ventral patch size) to select a male, their use of these signals differs both between them and among the social contexts examined in this study. Our inability to detect non-random behavior by yellow and orange females exposed to yellow and blue males may not necessarily reflect a lack of preference by females in these contexts. Rather, for yellow females at least, yellow males were preferred by yellow females in 83% of trials in this social context. This suggests a hierarchy in yellow female behavior: associate with same-colored males when they are available and, if not, associate with males having a smaller ventral patch. And, it may be that orange females use additional signals derived from other male traits as mate choice criteria, such as body size [43]. Because we controlled for body size in selecting the dyads, we are unable to detect preference for this trait by females. Body size influences both the outcome of male territorial contests [56] and female choice experiments in other lizard species [43]. For orange females, preference for larger-sized males would complement our findings that this morph prefers dominant males, but more work is needed to address this consideration.

Unlike dewlap color or ventral patch size, the role of femoral pore secretions in the mating behavior of *U. ornatus* is unclear. Previous work suggests that both sexes are capable of differentiating between conspecific femoral pore secretions and those produced by themselves [32]. We show that both chemical and dewlap color signals may elicit similar patterns of female behavior, suggesting that femoral pore chemical secretions also differ among male *U. ornatus* morphs. Yellow females discriminated among yellow and orange male morphs using chemical signals alone. Orange females behaved randomly with respect to male chemical signals. However, we did observe that orange females associated with a yellow male in 63% of all Y-O dyad trials in the chemical experiment. Male femoral pore secretions may signal individual quality, competitive ability, and dominance status to a conspecific [57], and consequently may be important for guiding female mating decisions [27], [30], [45], [58]. Moreover, the evolutionary maintenance of both visual and chemical signals in intersexual communication may be favored if they invoke redundant behavioral responses by a female [29]. Male *U. ornatus* chemical secretions may therefore be under selection to covary with the reproductive behavior differences that characterize their color polymorphism [31]. Our observation of similar patterns of female behavior with respect to male dewlap color and femoral pore secretions in these experiments provides some initial support for this hypothesis. Additional work is required to determine the degree of morph-specificity in the chemical profiles of male *U. ornatus* secretions [59]. An association between femoral pore secretions and male visual signals would provide further support that they are redundant in their role in female mate choice in *U. ornatus* [31], [59], [60].

### Conclusions

The combination of male morphs and non-random female preference within a population may enhance rates of sympatric divergence, provided that female preferences are fixed to discrete male traits and do not vary across different social contexts [4], [5]. An alternative hypothesis is that female preference is context-dependent and involves evaluating signals deriving from both discrete and condition-dependent male traits. These preferences, in conjunction with other mechanisms of selection acting in polymorphic systems (e.g., male-male competition and predation), might serve to contribute to the maintenance of discrete male phenotypes in a population. The results of [17] and our study support this hypothesis, and further suggest that female preference and male-male competition may be antagonistic in their effects on male ventral patch size, favoring the maintenance of overlap in patch size among all males [61]. Female mate selection may also be affected by male-male competition if the spatial dispersion of males in a population relative to a females’ preferred resources (e.g., oviposition sites) is non-random. Resources are abundant at our study site and the spatial dispersion of male morphs in the population is effectively random with respect to those resources (i.e., trees, Lattanzio & Miles, unpublished data). We therefore do not expect male competition to exert a significant influence on the probability of a female encountering a preferred male at this site. Alternatively, competition in resource-limited habitats may favor spatial reorganization within a population towards the monopolization of preferred resources by aggressive males [62]. In this scenario, male-male interactions will affect the context and probability of female encounters with each male morph in a population. An interaction between resource availability, morph status signals (visual and chemical), and female preference should have important implications for the maintenance and spatial dispersion of polymorphic phenotypes across a species’ range.

## Supporting Information

Figure S1 **Adult male (A) and female (B)** ***Urosaurus ornatus*** **color morphs.** In *U. ornatus*, both sexes exhibit a polymorphism in dewlap color which is fixed at maturity. Individual males also exhibit variation in the size of their blue ventral patch, but these size differences are not fixed to different morphs (*P*>0.2, see Results). Female *U. ornatus* do not express this patch.
(TIF)

Figure S2 **Percent reflectance of male** ***Urosaurus ornatus*** **dewlap and ventral patches.** For dewlap patches (A), lines are colored by male morph: blue, orange, or yellow. For ventral patches (B), lines are shaded by male morph: blue (n = 16, black line), orange (n = 8, dark grey line), or yellow (n = 5, light grey line). Male *U. ornatus* lizards included in this figure were captured at the same study site as males used in the current study. The spectral range shown in both graphs includes ultraviolet (300–399 nm, grey shaded region) and visible light (400–700 nm). Values used to construct these graphs are mean ±1.0 standard error (SE) percent reflectance at 10-nm intervals.
(TIFF)

Raw Data S1 **Female preference experiment, male morphology, and Figure S2 databases.**
(XLSX)
