# Supplementary figures and images for: Preference for Male Traits Differ in Two Female Morphs of the Tree Lizard, Urosaurus ornatus
Source: PLoS One. 2014 Jul 17;9(7):e101515. doi: 10.1371/journal.pone.0101515 (PMC4102484; doi:10.1371/journal.pone.0101515)

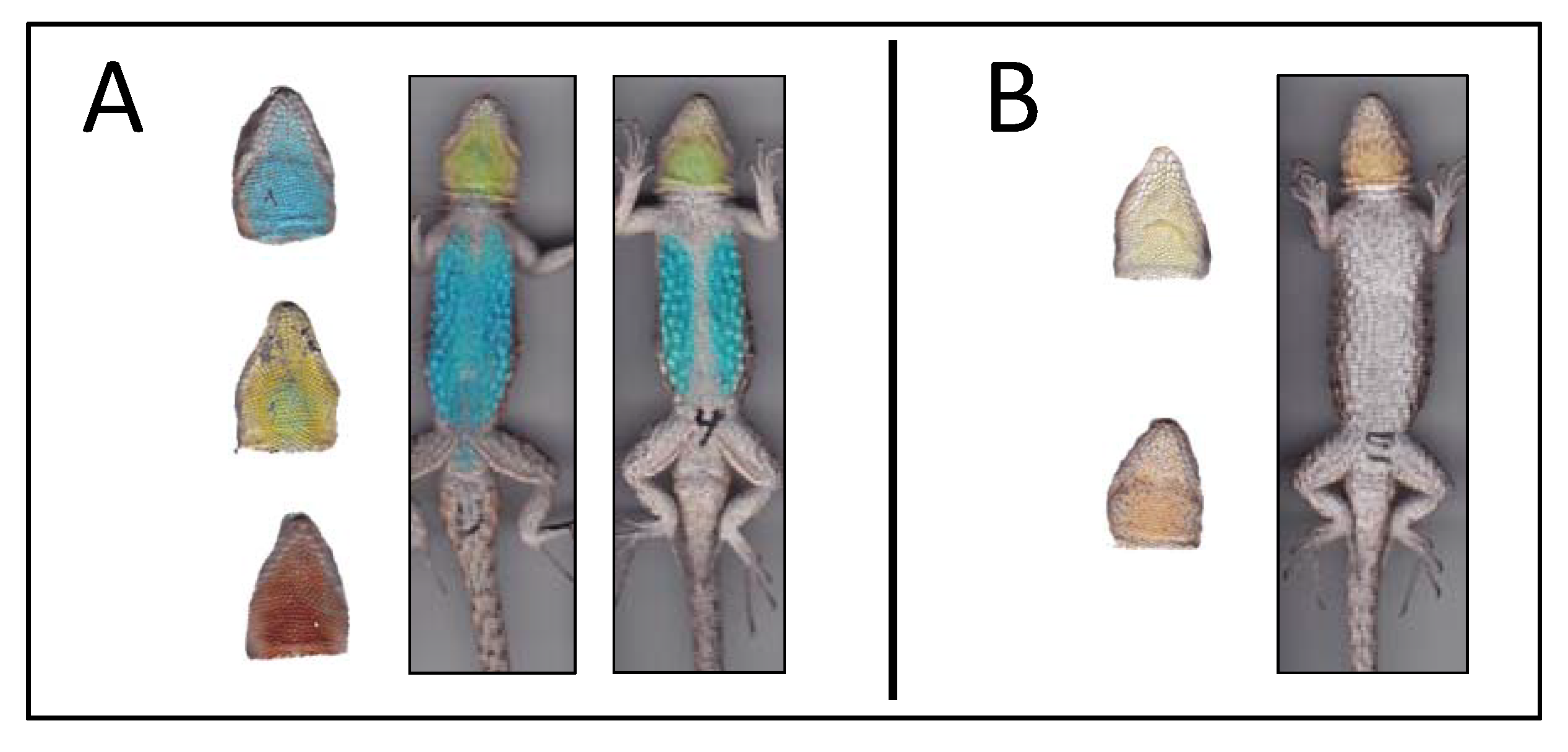

Supplement: Figure S1 — Adult male (A) and female (B) Urosaurus ornatus color morphs. In U. ornatus, both sexes exhibit a polymorphism in dewlap color which is fixed at maturity. Individual males also exhibit variation in the size of their blue ventral patch, but these size differences are not fixed to different morphs (P>0.2, see Results). Female U. ornatus do not express this patch. (TIF) [file pone.0101515.s001.tif]

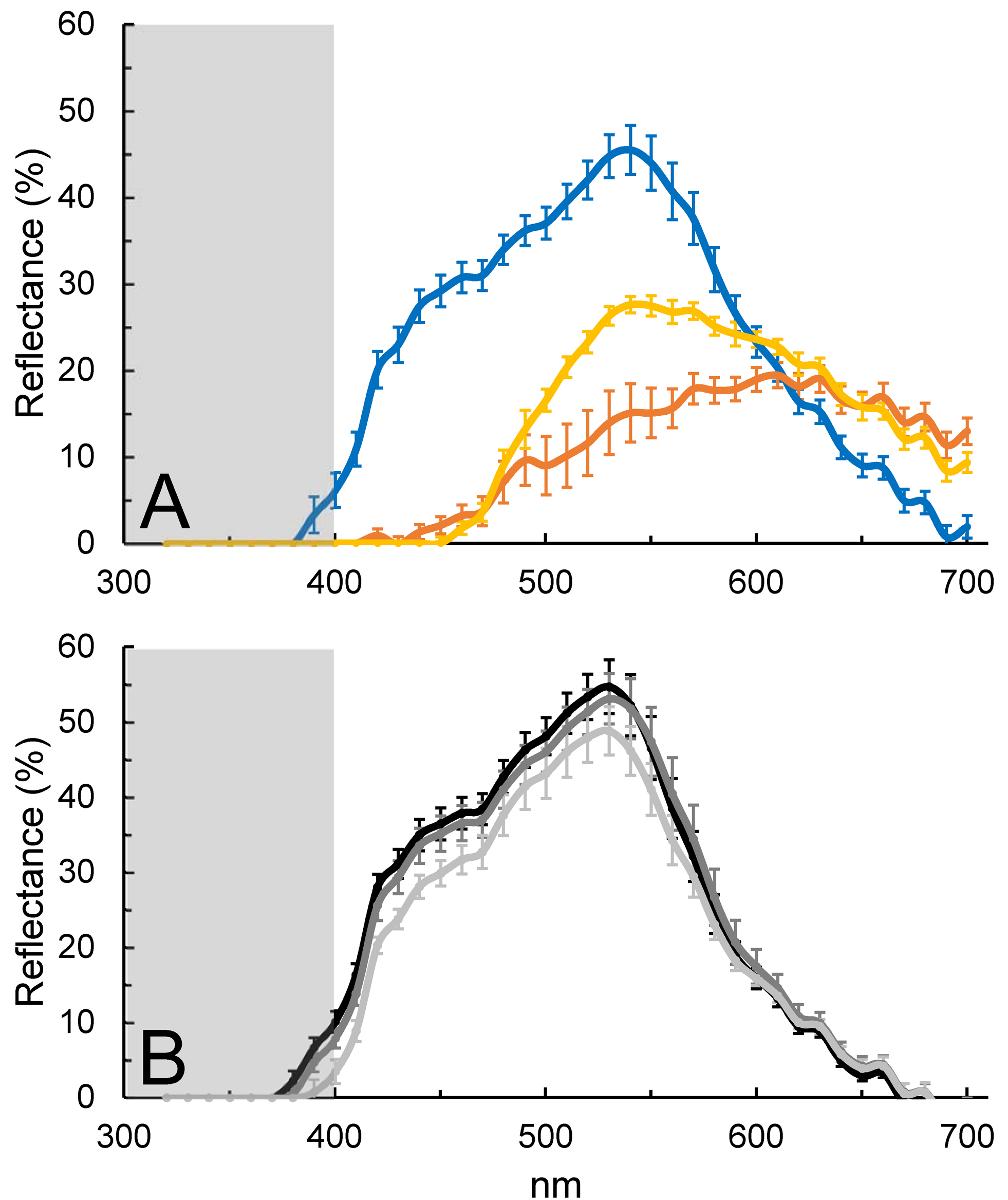

Supplement: Figure S2 — Percent reflectance of male Urosaurus ornatus dewlap and ventral patches. For dewlap patches (A), lines are colored by male morph: blue, orange, or yellow. For ventral patches (B), lines are shaded by male morph: blue (n = 16, black line), orange (n = 8, dark grey line), or yellow (n = 5, light grey line). Male U. ornatus lizards included in this figure were captured at the same study site as males used in the current study. The spectral range shown in both graphs includes ultraviolet (300–399 nm, grey shaded region) and visible light (400–700 nm). Values used to construct these graphs are mean ±1.0 standard error (SE) percent reflectance at 10-nm intervals. (TIFF) [file pone.0101515.s002.tiff]
